# Efficacy of Blood Sources and Artificial Blood Feeding Methods in Rearing of* Aedes aegypti* (Diptera: Culicidae) for Sterile Insect Technique and Incompatible Insect Technique Approaches in Sri Lanka

**DOI:** 10.1155/2017/3196924

**Published:** 2017-08-15

**Authors:** Nayana Gunathilaka, Tharaka Ranathunge, Lahiru Udayanga, Wimaladharma Abeyewickreme

**Affiliations:** ^1^Department of Parasitology, Faculty of Medicine, University of Kelaniya, Ragama, Sri Lanka; ^2^Molecular Medicine Unit, Faculty of Medicine, University of Kelaniya, Ragama, Sri Lanka; ^3^Dengue Mega Project, Faculty of Medicine, University of Kelaniya, Ragama, Sri Lanka

## Abstract

**Introduction:**

Selection of the artificial membrane feeding technique and blood meal source has been recognized as key considerations in mass rearing of vectors.

**Methodology:**

Artificial membrane feeding techniques, namely, glass plate, metal plate, and Hemotek membrane feeding method, and three blood sources (human, cattle, and chicken) were evaluated based on feeding rates, fecundity, and hatching rates of* Aedes aegypti*. Significance in the variations among blood feeding was investigated by one-way ANOVA, cluster analysis of variance (ANOSIM), and principal coordinates (PCO) analysis.

**Results:**

Feeding rates of* Ae. aegypti* significantly differed among the membrane feeding techniques as suggested by one-way ANOVA (*p* < 0.05). The metal plate method was identified as the most efficient and cost-effective feeding technique. Blood feeding rate of* Ae. aegypti* was higher with human blood followed by cattle and chicken blood, respectively. However, no significant difference was observed from the mosquitoes fed with cattle and human blood, in terms of fecundity, oviposition rate, and fertility as suggested by one-way ANOVA (*p* > 0.05).

**Conclusions:**

Metal plate method could be recommended as the most effective membrane feeding technique for mass rearing of* Ae. aegypti*, due to its high feeding rate and cost effectiveness. Cattle blood could be recommended for mass rearing* Ae. aegypti*.

## 1. Background

Dengue is the most rapidly spreading mosquito-borne viral disease in the world, which is mainly transmitted by* Aedes aegypti* mosquitoes [1]. During 2016, 54,364 suspected dengue cases have been reported to the Epidemiology Unit, Sri Lanka, from all over the island indicating the highest ever number of cases per year. Approximately, over 49% of dengue cases have been reported in 2016 from the Western province, Sri Lanka [2]. Absence of effective drugs and vaccines for dengue virus remains as the major issue in elimination of dengue from the country. Therefore, the main focus of the disease controlling entities is to control the vector densities of dengue, thereby restricting the transmission of the virus [3].

In Sri Lanka, current vector controlling approaches focus on environmental management, chemical control, and biological control. In view of the problems associated with conventional mosquito control, such as resistance and health effects, new vector control approaches such as sterile insect technique (SIT), incompatible insect technique (IIT), and use of transgenic mosquitoes have been evolved recently [4, 5]. However, these methods require mass rearing of mosquitoes for laboratory experiments and field based trials.

Nutrition is one of the main factors to be considered in mass rearing of mosquito vectors. Anautogenous female mosquitoes require protein from blood to develop eggs [6]. Laboratory studies on mosquito biology and vector-parasite interactions largely require artificial blood delivery systems to feed mosquitoes. Therefore, studies related to effective blood meal sources and artificial blood feeding techniques are essential in establishing laboratory colonies of mosquito vectors [7].

Most of the earlier studies on mosquito rearing relied on human hosts or live animal hosts [8–10]. Currently, live animals such as guinea pigs and rodents are more commonly used. Several different artificial feeders have been developed for feeding of mosquitoes; some are simple; some are more complex.

Various types of membranes are being used in artificial membrane techniques, such as animal tissue, Parafilm-M® films, and collagen membranes [11]. The cattle collagen sausage-casing membrane and Parafilm-M are inexpensively mass customized with quality control. The temperature of the blood meal is generally maintained at 37–39°C to attract the mosquitoes to settle [12]. Hemotek membrane feeders (Discovery Workshops, Accrington, UK) use an electric heating element to maintain the temperature of the blood meal [13, 14]. The glass feeder has an outer area with circulating warm water and an inner chamber into which the blood is poured [15, 16]. However, some of these methods are expensive, time consuming, subjective to regulation, and inspection, thereby limiting their usage in many laboratory settings [7]. On the other hand, some of these facilities are not affordable for some laboratories due to limitations of funds to acquire highly sophisticated apparatus and to bear maintenance cost, especially in the developing countries. Therefore, it is important to develop an inexpensive, convenient, and effective artificial membrane blood feeding method that may also take animal welfare into consideration.

Hence, the objective of the present study was to evaluate the effectiveness of different artificial blood feeding methods and appropriate blood meal source/s for rearing of* Ae. aegypti* vectors under laboratory conditions in order to facilitate the experiments, which have been implemented on several novel vector control strategies in Sri Lanka. The findings of the current study may encourage laboratories in different institutes which conduct colony maintenance, vector-parasite interactions, and medical entomology related research by seeking inexpensive and effective artificial membrane feeding methods, along with available blood sources that yield the optimum growth and reproductive rates of medically important mosquitoes of their interests.

## 2. Methods

### 2.1. Establishment of* Ae. aegypti* Colony

A mosquito colony of* Aedes aegypti* was established from five blood-engorged wild females caught from the field (Ragama Medical Officer of Health area) and was maintained under a 12 : 12 (light : dark) cycle. Mosquitoes were reared using standard conditions to generate similar sized individuals. Adult mosquitoes were housed in 24 × 24 × 24 cm mosquito cages with mesh screening on top, provided with a 20% sugar solution and water ad libitum. The colony was maintained at 27 ± 2°C and 75 ± 5% humidity, providing human blood as the feeding source. A hand-held aspirator was used to access mosquitoes through a tighten opening in one side of the cage. The colony was maintained under the above laboratory settings at the Molecular Medicine Unit, Faculty of Medicine, University of Kelaniya. The offspring from 12th generation of the above colony was used for the current study.

Five-day old females were randomly placed in screen topped oviposition plastic cups (15 cm in diameter and 13.5 cm in depth) with inner side lined with filter paper and filled with approximately 150 ml of filtered tap water. Females were allowed to feed on cattle blood and the eggs laid by them were allowed to be hatched within 2-3 days after oviposition. The first instar larvae (*L*_1_) were transferred daily from the oviposition cups to plastic trays (40 × 25 × 6 cm), containing 2,000 ml of water and reared to the 4th instar (*L*_4_) larvae with a larval diet containing 50% tuna meal, 36% bovine liver powder, and 14% yeast [17]. The larval rearing trays were examined daily for pupae, which were removed and transferred to mosquito cages for adult emergence.

### 2.2. Evaluation of the Membrane Feeding Techniques

A batch containing 300 mosquitoes (with 1 : 1 male : female ratio) of* Ae. aegypti* in the same age (2-3 days old) was introduced in to 24 × 24 × 24 cm cages using a hand-held aspirator and fed with cattle blood using three different artificial membrane feeding techniques as described below. Blood samples were shaken for 30 seconds at every 30 minutes and the temperature was maintained at 37°C to minimize the sedimentation of blood throughout the experiment. After 2 hours of blood feeding, the number of female mosquitoes that successfully fed was recorded and mosquitoes that did not feed or only partially fed were discarded. The procedure was repeated 5 times for each blood feeding technique using the adult* Ae. aegypti* mosquitoes from the same cohort with similar age as described below.

### 2.3. Glass Plate Method

**Figure 1:**
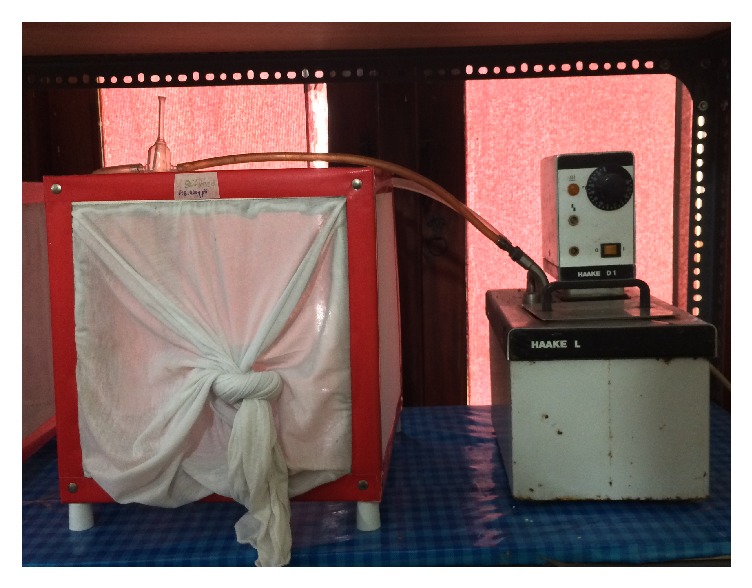
Setup of the glass plate method.

A circulating water bath was set to 37°C and was connected to holding containers via tubing. A Parafilm-M membrane was stretched across the bottom of the glass tube of the feeder (HAAKE, Model 000-3350), with a surface area of 7.07 cm^2^, and secured with a rubber band. Blood was added (3.0 ml) to the funnel of the glass feeder (Figure 1).

### 2.4. Metal Plate Method

**Figure 2:**
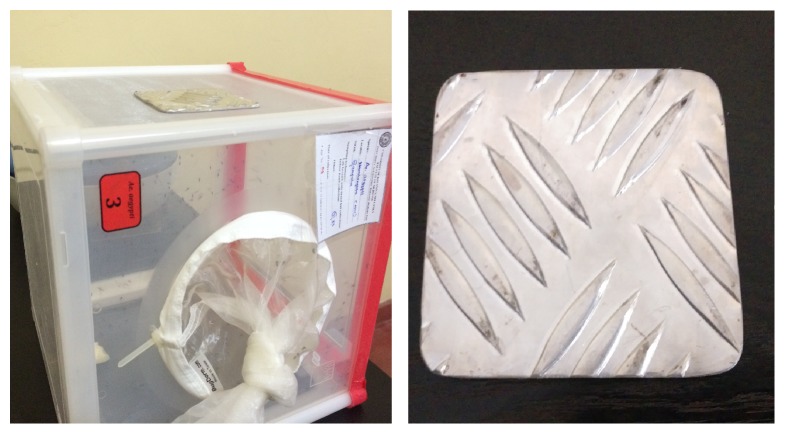
Metal plate and the setup of the metal plate method.

A square shaped metal plate with a rough surface (9 cm × 9 cm: width × length) was prepared. The rough surface of the developed metal plate was covered with a stretched Parafilm-M membrane leaving one side open. Blood was added (3.0 ml) into the space between the plate and the membrane, and the open side was carefully sealed. A small pillow (10 cm × 10 cm: width × length) filled with rice grains was heated up to 40°C using a microwave oven and was placed on top of the metal plate, while keeping blood filled surface down in order to maintain the temperature throughout the time of blood feeding (Figure 2).

### 2.5. Hemotek Membrane Feeding Method

**Figure 3:**
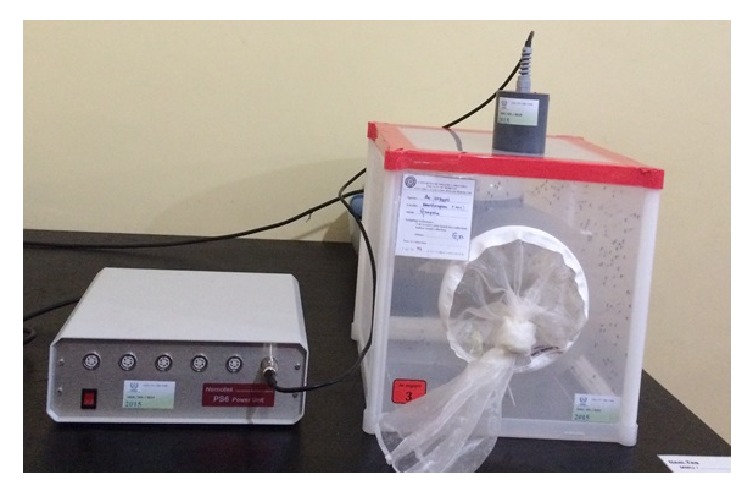
Setup of the Hemotek membrane feeding system.

A Parafilm-M membrane was stretched across the bottom of the Hemotek membrane blood feeder (PS-6 System, Discovery Workshops, Accrington, UK) having a surface area of 9.62 cm^2^. Blood (3.0 ml) was transferred into the Hemotek blood reservoir unit and the temperature was set to 37°C (Figure 3).

### 2.6. Evaluation of Blood Meal Success by* Ae. aegypti*

Three different blood meal sources, namely, human, cattle, and chicken, were taken for the study. Both cattle and chicken blood were purchased from a slaughter house (acquired from a healthy individual), which is operated in accordance with standard protocols under the supervision of Public Health Inspectors (PHI). Human blood was obtained from a healthy volunteer (<18 years) after obtaining the written consent from the donor. The blood was taken under sterile condition by a trained technical officer under supervision of a Medical Officer at the Faculty of Medicine, University of Kelaniya, Sri Lanka.

The blood samples were stored at 4°C after adding ethylene diamine tetraacetic acid (EDTA) as an anticoagulant (EDTA : blood = 1 : 100) until taken for experiments. Prior to the experimental trials, blood samples were kept at room temperature for 30 minutes and introduced to the female mosquitoes in holding containers using metal plate method as described above.

Batches of 300* Ae. aegypti* mosquitoes (with 1 : 1 male to female ratio) were fed with blood using metal plate method, as described above. After feeding of blood, the mosquitoes were transferred into screen topped oviposition plastic cups (15 cm in diameter and 13.5 cm in depth) with inner side lined with filter paper and filled with approximately 150 ml of filtered tap water.* Ae. aegypti* mosquitoes were allowed to mate naturally till two days after blood feeding within the container. After 2 days, the filter paper lining within each container was taken out and were allowed to dry for 7 days. The number of eggs on each paper was enumerated under a light microscope by trained personals along with accepted quality control procedures.

Subsequently, eggs were allowed to be hatched separately and the first instar larvae (*L*_1_) that emerged from each filter paper lining were enumerated, separately for 2 days after introduction of the egg papers into the hatching bottles. The whole procedure was repeated for 5 times for each blood source.

### 2.7. Data Interpretation and Statistical Analysis

Blood feeding rates of female mosquitoes were calculated for each membrane feeding method and blood meal source, separately. Fecundity of the female mosquitoes that were fed with different sources of blood was calculated as the number of eggs laid by the females. The oviposition rate for females was calculated as the percentage of eggs laid by fully engorged females, while fertility was calculated as the percentage of larvae that emerged after hatching as recommended by Clements, 1992 [6].

The SPSS (version 21) and Plymouth Routines in Multivariate Ecological Research version 6 (PRIMER 6) were devised to perform the statistical comparisons. In case of nonnormal distributions, normalization and square root transformation techniques in PRIMER 6 were employed before processing of data. Significance in the variations among blood feeding rates of three membrane feeding techniques and variations among blood feeding rates, fecundity, and egg hatching rate for each blood meal source were evaluated using General Linear Modelling followed by Tukey's HSD (Honest Significant Difference). Further, cluster analysis (with Bray Curtis resemblance), analysis of variance (ANOSIM), and principal coordinates (PCO) were used to investigate the effect of different blood sources on the investigated parameters cumulatively.

## 3. Results

### 3.1. Evaluation of Membrane Feeding Techniques

Mean number of female mosquitoes that fed from different feeding methods and the blood feeding rates of females for each membrane feeding technique are presented in Table 1, along with the results of General Linear Modelling. The highest blood feeding rate of* Ae. aegypti* was identified from metal plate method (98.67%, *n* = 296) followed by Hemotek method (90.33%, *n* = 271) and glass plate method (83.67%, *n* = 251). Therefore, metal plate method can be identified as the most efficient blood feeding method for* Ae. aegypti* (*p* < 0.05 at 95% level of confidence).

### 3.2. Evaluation of Blood Source Success of* Ae. aegypti*

Mean percentage of female mosquitoes that fed with different sources of blood, fecundity, their egg laying efficiency, and egg hatching rate are presented in Table 2 along with the results of General Linear Modelling. Among the three blood meal sources tested, human blood was identified as the most preferred blood source (Table 2) by* Ae. aegypti* followed by cattle and chicken blood in terms of both number of blood fed females and blood feeding rate (*p* < 0.05 at 95% level of significance). However, the fecundity, egg laying efficiency, and eggs hatching rate were not indicating any significant differences among human and cattle origin blood sources according to the results of General Linear Modelling followed by Tukey's pair wise comparison (Table 2).

**Figure 4:**
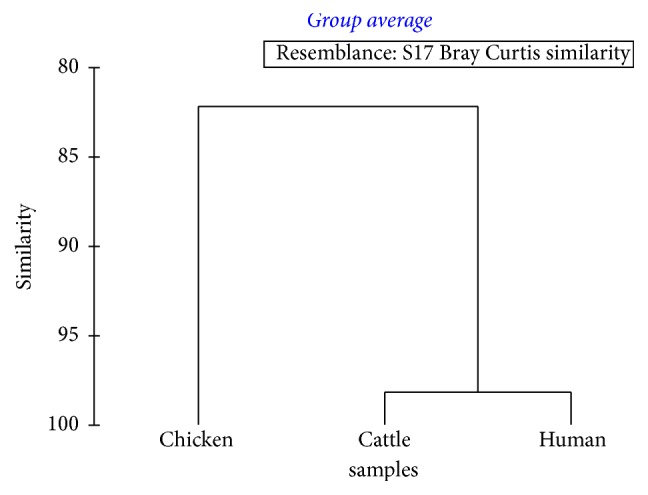
Dendrogram for the cluster analysis of blood source success by* Ae. aegypti*.

**Figure 5:**
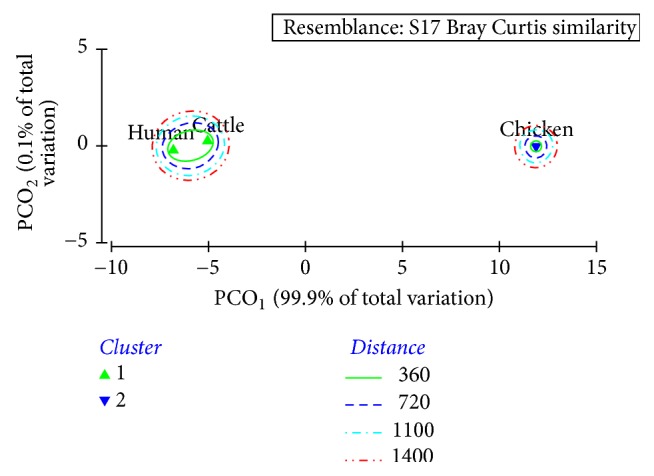
Ordination of the different blood sources on PCO_1_ and PCO_2_ scores of PCO analysis in terms of success of* Ae. aegypti*.

The overall clustering status of different blood sources in terms of blood feeding rate, fecundity, egg laying efficiency, and egg hatching rate of* Ae. aegypti* is illustrated in Figure 4. As suggested by the Bray Curtis similarity clustering, both human and cattle blood indicated a high similarity of 98.17%, while chicken blood indicated a dissimilarity of 17.81% with other two sources based on the cumulative effect of blood feeding rate, fecundity, egg laying efficiency, and eggs hatching rate. The global *R* value of 0.97 gained for the analysis of similarities (ANOSIM) also confirmed the above observation at a significance level of 5%. Meanwhile, both PC_1_ and PC_2_ axes of the principal coordinates (PCO) that accounted for the total variation (100%) of the blood source success of* Ae. aegypti* (Figure 5) suggested the emergence of two major clusters as human and cattle blood together, while chicken blood remained isolated. Hence, the results of one-way ANOVA are further confirmed by the cluster and PCO analytical procedures.

## 4. Discussion

Many researches are involved in developing innovative vector control approaches along with conventional vector control methods. However, these approaches need laboratory experiments and artificial rearing of vectors in mass scale. In order to achieve this, it is essential to provide necessary conditions for them to grow, rather in an anthropogenic environment. Many epidemiologically important issues revolve around the feeding behaviour of vector mosquitoes [18]. Selection of a suitable blood meal source and cost-effective technique are the main challenges at insectary setup. Membrane blood feeding system as an artificial feeder to blood-feed* Aedes* mosquitoes is unique and suitable for multiple purposes. It is important to develop an inexpensive, convenient, and effective artificial membrane blood feeding technique that takes animal welfare into consideration.

Artificial feeding systems that have been developed differ with respect to the composition of the meal, the nature of the membrane, and the method of temperature regulation [14]. Several types of liquid diets including goose, chicken, bovine, pig, mice, rat, gerbil, hamster, rabbit, nonhuman primate blood types, and artificial diets are being used for the purpose of blood feeding mosquitoes [16].

A previous study with blood fed* Ae. aegypti* indicated the highest fecundity by avian blood than mammalian blood [19]. Conversely, Suleman and Shirin [20] showed that mammalian blood results in greater fecundity than avian blood in* Culex quinquefasciatus*. These studies demonstrate variations in fecundity between species fed with different blood sources [19–21]. Despite fecundity differences, no variation was seen in fertility (number of larvae hatched from eggs) for* Cx. quinquefasciatus* provided with different blood sources [20].

According to the available literature,* Ae. aegypti* prefers mammalian hosts [22] and may preferentially feed on humans, even in the presence of alternative hosts [23]. It is evidenced the* Aedes* mosquitoes feed multiple times during one gonotrophic cycle [24], which may facilitate the disease transmission. However, the host preference of* Ae. aegypti* in Sri Lanka is not well understood, since there is no study conducted to evaluate the hematophagous tendencies among wild population. Therefore, the studies on above aspects should be given high priory in Sri Lanka preceding to the release of transgenic or sterile mosquitoes in controlling dengue vectors.

The present study indicated that the blood meal source affects feeding rates and reproduction in colonized* Ae. aegypti* under the laboratory conditions. Mosquitoes fed on human blood demonstrated higher feeding rates, fecundity, oviposition, and fertility rates than those fed with other two (cattle and chicken) blood meal sources. However, obtaining human blood for insectary colony maintenance is problematic due to several ethical and safety issues. In addition, human blood is very scares, since there are limited donors. Human blood stored at the blood banks mainly provides blood for medical emergencies rather than experimental work at insectaries.

Therefore, selection of a blood meal source which is fairly efficient and readily available compared to human blood is essential in rearing. Some studies evidenced that the chicken blood contains nucleated blood cells, while mammalian blood contains anucleated cells, which may influence fecundity if nucleated cells contain more nutrition [20, 25]. However, the present study revealed the lowest fecundity from chicken blood.

As there were no significant differences among mosquitoes fed with cattle and human blood in terms of fecundity, oviposition rate, and fertility, the cattle blood may be suggested to be used as an appropriate blood meal source for rearing of* Aedes* mosquitoes, even though there was a difference between the numerical values with human blood. On the other hand, cattle blood could be easily obtained from farms or slaughter houses that are operated adhering to the government regulations with concerning animal welfare at very low costs or sometimes with no cost. In addition, blood related infections that can be transmitted through cattle blood to the workers accidentally at insectaries will be minimal compared to the use of human blood. Therefore, cattle blood could be recommended as the most convenient blood source to be used under local settings with minimal health risks to coworkers.

The results of the present study demonstrate that the blood meal source may influence physiological processes related to fecundity and fertility in* Ae. aegypti*. However, further studies are needed to address the extent to which these effects alter interactions between mosquitoes and blood meal sources.

Among the artificial membrane feeding methods, the metal plate method was identified as the most efficient feeding method. Since both Hemotek and glass plate techniques require expensive machinery and electricity, use of metal plate method can be suggested as a cost-effective method to achieve artificial blood feeding for hematophagous insects rearing at laboratory settings.

## 5. Conclusion

Metal plate feeding technique could be recommended as the most effective membrane feeding technique for mass rearing of* Aedes aegypti* compared to glass plate and Hemotek membrane feeding methods, due to its high feeding rate and cost effectiveness. As there were no significant differences among mosquitoes fed with cattle and human blood in terms of fecundity, oviposition rate, and fertility, the cattle blood may be suggestive to use as an appropriate blood meal source for rearing of* Aedes* mosquitoes out of other blood meal sources tested (human and chicken), even though there was a difference between the numerical values with human blood.

## Figures and Tables

**Table 1:** Mean number of female mosquitoes that fed from different feeding methods, blood feeding rate along with the results of General Linear Modelling (number of replicates [*n*] = 5).

Feeding technique	Number of females	Number of blood fed	Blood feeding rate (%)
Metal plate	300	296 ± 2.33 (293.67–298.33)	98.67 ± 0.77^a^ (97.90–99.44)
Hemotek membrane feeding method	300	271 ± 2.33 (268.67–273.33)	90.33 ± 0.77^b^ (97.90–99.44)
Glass plate	300	251 ± 5.29 (245.71–256.29)	83.67 ± 1.76^c^ (81.91–85.43)

*Note*. Values are mean ± SE, range in parenthesis. Different superscript letters in a column show significant differences (*p* < 0.05) as suggested by General Linear Modelling followed by Tukey's HSD (Honest Significant Difference) at 95% level of significance.

**Table 2:** Mean percentage of female mosquitoes that fed with different blood types, blood feeding rates, their egg laying efficiency, egg hatching rate, and the results of General Linear Modelling (number of replicates [*n*] = 5).

Type of blood	Number of females	Number of blood fed females	Blood feeding rate (%)	Fecundity	Egg laying efficiency/oviposition rate	Number of eggs hatched	Egg hatching rate (%)
Cattle	300	270 ± 2.89^a^ (267.11–272.89)	89.67 ± 0.96^a^ (88.71–90.63)	4196 ± 39.10^a^ (4156.90–4235.10)	15.54 ± 0.31^a^ (15.23–15.85)	3904 ± 26.30^a^ (3877.70–3930.30)	93.04 ± 0.25^a^ (92.79–93.29)
Human	300	292 ± 2.93^b^ (289.07–294.93)	97.33 ± 0.99^b^ (96.34–98.32)	4336 ± 45.25^a^ (4290.75–4381.25)	14.85 ± 0.39^a^ (14.46–15.24)	4064 ± 40.9^a^ (4023.10–4104.9)	93.73 ± 0.75^a^ (92.98–94.48)
Chicken	300	239 ± 2.73^c^ (236.27–241.73)	79.67 ± 0.91^c^ (78.76–80.58)	3034 ± 34.90^b^ (2999.10–3068.9)	12.69 ± 0.28^b^ (12.41–12.97)	2681 ± 33.70^b^ (2647.30–2714.7)	88.39 ± 2.65^a^ (85.74–91.04)

*Note*. Values are mean ± SE, range in parenthesis. Different superscript letters in a column show significant differences (*p* < 0.05) as suggested by General Linear Modelling followed by Tukey's HSD (Honest Significant Difference) test, at 95% level of significance.
